# ITPR1 Deletion in a Patient With Sensory Ataxic Neuropathy and Sjögren Syndrome

**DOI:** 10.1111/jns.70083

**Published:** 2025-12-01

**Authors:** Saif Haddad, Roy Poh, Jason Hehir, James M. Polke, Julian Blake, Mary M. Reilly

**Affiliations:** ^1^ Centre for Neuromuscular Diseases, Department of Neuromuscular Diseases UCL Queen Square Institute of Neurology London UK; ^2^ Neurogenetics Laboratory National Hospital for Neurology and Neurosurgery and North Thames Genomics Laboratory Hub London UK; ^3^ Department of Clinical Neurophysiology Norfolk and Norwich University Hospital Norwich UK

**Keywords:** CMT, neurogenetics, SCA15, sensory ataxic neuropathy, spinocerebellar ataxia

## Abstract

**Background:**

Sensory ataxic neuropathies (SAN) are rare large fibre sensory neuropathies characterised by progressive sensory loss and ataxia. They may be inherited or acquired. When inherited they are more commonly seen as part of a broader syndrome involving cerebellar ataxia or mitochondrial dysfunction. Isolated inherited SAN are rare, and the causes are limited, including *RFC1* expansions (CANVAS), *POLG* variants and variants in *COX20* and *RNF170*. Spinocerebellar ataxia type 15 (SCA15), caused by deletions in the ITPR1 gene, is another potential genetic cause of SAN, as peripheral neuropathy is commonly associated with various spinocerebellar ataxias.

**Methods:**

A 35‐year‐old female who presented with an asymmetrical upper limb predominant sensory ataxic neuropathy which had initially been treated as a vasculitic neuropathy at her local hospital, underwent further evaluation.

**Results:**

Genetic analysis identified an approximately 6.5 Mb terminal deletion of chromosome 3p, including the *ITPR1* gene.

**Conclusions:**

This case report adds to the growing body of literature on ITPR1‐related diseases, highlighting the phenotypic variability of ITPR1 and identifying it as a potential cause of isolated SAN. While a connection to Sjögren's syndrome cannot be excluded, the role of *ITPR1* as the primary cause remains the focus. We propose that the neuropathy community consider screening SAN patients for *ITPR1* deletions, as additional cases may help clarify its clinical significance.

## 1 Background and Aims

Sensory ataxic neuropathies (SAN) are a rare subtype of inherited neuropathy. They are often part of a complex syndrome including the cerebellar ataxias or mitochondrial disorders. The genetic causes of a pure or predominant SAN are limited to a small number of genes; intronic expansions in *RFC1* cause CANVAS (cerebellar ataxia, neuropathy and vestibular areflexia syndrome) but can present as a pure SAN, similarly, recessive variants in the nuclear mitochondrial gene *POLG* classically cause SANDO (sensory ataxic neuropathy, dysarthria and ophthalmoplegia) but also can present with isolated SAN. Rarely variants in *COX20* and *RNF170*, reported in only a few families, can cause SAN [1]. We previously reported PNPT1 mutations as a novel genetic cause of SAN, expanding the spectrum of known genetic factors involved in this rare condition [2].

Spinocerebellar ataxia 15 (SCA15) is an adult‐onset autosomal dominant cerebellar ataxia, primarily caused by deletions in the inositol 1,4,5 triphosphate receptor type 1 (ITPR1) gene [1, 3]. ITPR1 plays a crucial role in calcium release from the endoplasmic reticulum, which is abundant in Purkinje cells. SCAs represent a clinically and genetically heterogeneous group of progressive disorders [3]. Peripheral neuropathy is frequently observed in spinocerebellar ataxia (SCA), but subtype‐specific neuropathic features and subclinical manifestations have rarely been evaluated [3, 4, 5, 6].

We present a rare case of a female patient with progressively worsening SAN, initially treated for vasculitis, but later found to have an ITPR1 gene deletion. While a potential association with Sjögren's syndrome was considered, the genetic finding suggests a distinct cause, warranting further exploration of ITPR1 variants in undiagnosed SAN cases.

## 2 Case Report

The proband was a 35‐year‐old female with no past medical history at first presentation. She reported a normal birth and was asymptomatic until the age of 35 years. There was no family history of neurological disease. She presented at her local hospital with numbness and shock‐like bursts of pain in the first three digits accompanied by worsening dexterity in the right hand. She was diagnosed with carpal tunnel syndrome and had two carpal tunnel release surgeries with no improvement in her symptoms. At the age of 38 years, the patient reported increasing numbness in both hands with functional difficulty as well as intermittent episodes of pain in the feet. She underwent multiple tests at her local hospital. Initial neuropathy screening revealed a normal full blood count, renal profile, erythrocyte sedimentation rate, C‐reactive protein, vitamin B12, folate, homocysteine and methylmalonic acid levels. The antinuclear antibody (ANA) was negative; however, the extractable nuclear antigen (ENA) panel was positive, showing the presence of anti‐Ro and anti‐LA antibodies along with elevated rheumatoid factor (RF). Nerve conduction studies (NCS) revealed a sensory neuropathy in the right upper limb with absent ulnar and radial sensory nerve action potential (SNAP). Magnetic resonance imaging (MRI) of the brain and cervical spine showed no abnormalities. A right sural biopsy revealed axonal loss without any evidence of inflammation, including no signs of vasculitis, along with clusters of axonal regeneration, indicating ongoing nerve damage and attempts at repair.

Her local team still felt this was likely to be a vasculitic neuropathy due to the pain and the patchiness and treated her with cyclophosphamide and an initial high dose and subsequent reducing course of prednisolone over 6 months. Her neuropathy did not improve, and she was referred to our tertiary centre for further evaluation. At this stage, the area of numbness had extended to the right upper forearm and right distal leg. She also described a clear episode of stabbing pains between T5 and T10 in the right anterior abdomen for 2 weeks followed by numbness. Otherwise, since onset she did not report any weakness in the upper or lower limbs. There were no symptoms above the neck, no sicca, joint involvement or systemic symptoms. On examination, her gait was steady, but she had a positive Romberg's sign. Cranial nerves were unremarkable. There was clear pseudoathetosis in her right arm. Tone and power were normal throughout except for minimal weakness in the right abductor digit minimi. Reflexes were globally absent, except for the right biceps reflex. Plantar responses were down‐going bilaterally, and no cerebellar signs were present. Sensory examination revealed asymmetric. Pinprick loss to the top of the right arm and distally in the lower limbs bilaterally. Vibration sense was reduced to the sternum in the right arm, and proprioception was impaired at the right wrist. The remainder of the sensory examination was normal.

Routine investigations, as well as additional specialist tests, were carried out which were unremarkable. However, ANA revealed a speckled 1:15120, positive ENA including anti‐Ro and anti‐LA and an elevated RF of 25.2 (normal 0–14). Cerebrospinal fluid was completely unremarkable including normal protein and cytology. NCS revealed a sensory neuropathy with absent median, ulnar and radial sensory amplitudes in the right upper limb and reduced superficial peroneal amplitude (3 μV) in the right lower limb as well as absent left radial amplitude and reduced left ulnar amplitude (5 μV) (Table 1) Motor conduction study and needle electromyography (EMG) were normal (Table 1). MRI of the brain, whole spine including brachial and lumbosacral plexuses was normal. A fluorodeoxyglucose positron emission tomography (FDG PET) scan did not show any avid disease. A lip biopsy was performed which showed a focus of lymphocytes consistent with a diagnosis of Sjogren's disease. A Schirmer test was normal. A right dorsal ulnar biopsy was also performed which showed chronic axonal neuropathy with subtotal loss of large myelinated fibres, relative preservation of small fibres, evidence of regeneration and endoneurial fibrosis, but no significant demyelination or inflammatiosn. In view of a possible diagnosis of Sjogren's associated neuropathy and previous failure to respond to steroids, she was given a trial of intravenous immunoglobulins (IVIG) (2 g/kg monthly × 3 courses) without any objective improvement.

**TABLE 1:** Nerve conduction studies.

Sensory NCS		
Nerve	Amp (μV)	Amp (μV)
	Right	Left
Radial (forearm‐wrist)	Absent	Absent
Median (F2‐wrist)	Absent	ND
Median (F3‐Wrist)	Absent	8
Ulnar (F5‐wrist)	Absent	5
Superficial peroneal (calf‐ankle)	3	10
Sural (calf‐ankle)	Biopsy^a^	11

Motor NCS			
Nerve	DML	Amp	CV
	(ms)	(mV)	(m/s)
Right Median (SE on APB)	3.5	8.2	49
Right Ulnar (SE on ADM)	2.6	5.0	59
Right Common Peroneal (SE on EDB)	3.9	4.2	45

Abbreviations: ADM, abductor digiti minimi; Amp, amplitude; APB, abductor pollicis brevis; CV, conduction velocity; DML, distal motor latency; EDB, extensor digitorium brevis; NCS, nerve conduction studies; ND, not done.

^a^ Nerve conduction studies for patients.

Over the next 10 years, she reported gradual progressive difficulty with walking, balance and fine hand movements. At age 52, she reported increasing left‐sided involvement. Examination showed subtle head titubation and an ataxic gait. As previously noted, there was a widespread reduction in pinprick sensation on the right side, but this had extended in a patchy distribution to the left arm and left mid‐thigh of the leg. The remainder of the examination remained unchanged. Consequently, IVIG was restarted. Although she reported a subjective improvement, there was no objective improvement and IVIG was stopped.

**FIGURE 1:**
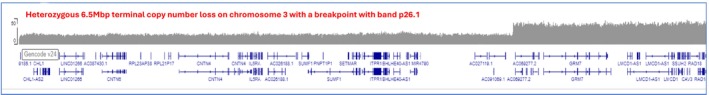
The integrative genomics viewer (IGV) file reveals a heterozygous deletion encompassing *ITPR1* and several neighbouring genes. Among them, *ITPR1* is the most likely cause of the phenotype, as similar deletions have been associated with autosomal dominant SCA15.

Given the slow progression and lack of evidence for acquired causes, genetic testing was performed. Panels of genes associated with neuropathy and mitochondrial disorders were sequenced by whole genome sequencing. PCR was performed to exclude intronic expansions in *RFC1* and whole mitochondrial sequencing was performed. Analysis of her whole genome revealed a heterozygous 6.5Mbp terminal copy number loss on chromosome 3 with a breakpoint at band p26.1 (Figure 1). Parental testing was not available.

The deletion encompasses approximately 40 named genes, including 13 protein‐coding genes, four of which have established disease associations: *TRNT1*, *CRBN*, *SUMF1* and *ITPR1*, of which only *ITPR1* causes a dominant condition; the others are associated with recessive conditions not relevant to this patient's phenotype. Importantly, the deletion includes the entire *ITPR1* gene (Figure 1).

Given ITPR1's well‐established role in autosomal dominant SCA15, along with previously described cases with cerebellar ataxia and peripheral neuropathy and supporting evidence from our clinical experience with other genes—such as PNPT1‐related ataxia and CANVAS causing SAN—we consider a deletion of ITPR1 to be a plausible explanation for the patient's neurological phenotype.

The deleted interval also includes *SETMAR, SUMF1, PNPT1P1, CBFB, IL5RA* and *ARL8B*, genes implicated in intracellular signalling, immune function, gene expression and cellular maintenance. However, none of these have strong individual associations with the observed clinical features. While *SUMF1* has occasionally been co‐deleted with *ITPR1* in other SCA15 cases, deletions of *SUMF1* alone have not been linked to this condition.

A key study by Moghadasi et al. [7] reported a four‐generation family carrying a ~2.9 Mb overlapping 3p26.3 deletion—including adjacent genes but not *ITPR1*—in multiple unaffected individuals. This finding suggests that deletion of these more distal genes may be insufficient to cause disease in isolation.

Together, these findings support *ITPR1* as the primary pathogenic gene in our patient's deletion, while other genes within the deleted region are likely to have minimal or no clinical impact in this context.

## 3 Discussion

We report a patient with a slowly progressive SAN, initially treated for suspected inflammatory neuropathy with cyclophosphamide, prednisolone and IVIG. Although Sjögren's was considered, genetic testing identified an ITPR1 deletion, suggesting a potential cause, though not definitive.

SCAs are a rare neurodegenerative disease that is clinically and genetically heterogenous [1]. To date, there are 48 reported SCAs. SCA15 is a degenerative, adult‐onset autosomal dominant cerebellar ataxia, primarily caused by deletions in the ITPR1 gene. It was initially described by Story et al. in an Australian family with pure cerebellar ataxia with deletions of exons 1–10 of ITPR1 and half of the SUMF1 gene. ITPR1 is crucial for intercellular calcium signalling, facilitating the release of calcium from the endoplasmic reticulum into the cytosol. Activation of the tetrameric channel is initiated as four molecules of inositol 1,4,5‐triphosphate (IP3) bind to each subunit of the IPC3 receptor channel. Pathogenic heterozygous missense variants also cause SCA29 (congenital onset). Gillespie syndrome, a rare disorder characterised by a triad of aniridia, cerebellar ataxia and intellectual disability which can be autosomal dominant or autosomal recessive is also associated with nonsense, missense and microdeletion varaints [5].

To date, there are approximately 80 reported cases of SCA15. The age of onset ranges from 20 to 60 years old (mean age, 39.6 years old). Tipton et al. provide a comprehensive review of 60 SCA15 patients including their phenotypic and genotypic characteristics [3]. Common phenotypic presentations included gait ataxia (88.3%) followed by dysarthria (73.3%), nystagmus (73.3%) and limb ataxia (71.7%). Hara et al. identified two Japanese families with autosomal dominant cerebellar ataxia with a suggestion of a mild peripheral neuropathy including reduced vibration and absent reflexes with a 414‐kb deletion including the entire ITPR1 gene and exon 1 of the SUMF1 gene and a missense mutation Pro1059Leu in ITPR1 in the two families respectively. Although conduction velocities were reported as normal, a full set of neurophysiological data was not available [5, 6].

The ITPR1 deletion is a possible cause of the SAN phenotype in our patient, as it has been previously associated with SCA15 with a suggestion of an associated neuropathy. The mild head titubation observed in our patient may represent early cerebellar involvement, although a repeat MRI remains normal at this stage. Other SCAs such as PNPT1‐related ataxia and CANVAS have also been associated with SAN.

In conclusion, ITPR1‐related deletions are a rare cause of cerebellar ataxia. While the patient carries a 6.5 Mb deletion, her SAN is likely attributable to the loss of ITPR1. This case adds to the growing list of phenotypes associated with ITPR1 and underscores the potential for SAN presentation. It also emphasizes the importance of considering genetic causes in slowly progressive neuropathies, particularly in cases with patchy onset or those unresponsive to treatment in the context of presumed inflammatory neuropathies. Although the history raised the possibility of Sjögren's, the identification of an ITPR1 deletion suggests a genetic cause, though its definitive relevance remains uncertain. We recommend that the neuropathy community consider screening undiagnosed SAN patients for ITPR1 variants, as additional cases may surface.

## Author Contributions

S.H. and M.M.R. contributed to the conception and design of the study. S.H., R.P., J.M.P., J.H., J.B. and M.M.R. contributed to the acquisition and analysis of data. S.H. and M.M.R. contributed to drafting the text or preparing the figures.

## Consent

Patient consent was obtained.

## Conflicts of Interest

The authors declare no conflicts of interest.

## Data Availability

The data that support the findings of this study are available from the corresponding author upon reasonable request.
